# Parvovirus B19 and Human Herpes Virus 6B and 7 Are Frequently Found DNA Viruses in the Human Thymus But Show No Definitive Link With Myasthenia Gravis

**DOI:** 10.1093/infdis/jiae600

**Published:** 2024-12-05

**Authors:** Kirsten Nowlan, Leo Hannolainen, Irini M Assimakopoulou, Pia Dürnsteiner, Joona Sarkkinen, Santeri Suokas, Lea Hedman, Pentti J Tienari, Klaus Hedman, Mikael Niku, Leena-Maija Aaltonen, Antti Huuskonen, Jari V Räsänen, Ilkka K Ilonen, Mikko I Mäyränpää, Johannes Dunkel, Sini M Laakso, Maria Söderlund-Venermo, Maria F Perdomo, Eliisa Kekäläinen

**Affiliations:** Translational Immunology Research Program, University of Helsinki, Helsinki, Finland; Department of Bacteriology and Immunology, University of Helsinki, Helsinki, Finland; Department of Virology, University of Helsinki and Helsinki University Hospital, Helsinki, Finland; Department of Virology, University of Helsinki and Helsinki University Hospital, Helsinki, Finland; Translational Immunology Research Program, University of Helsinki, Helsinki, Finland; Department of Bacteriology and Immunology, University of Helsinki, Helsinki, Finland; Translational Immunology Research Program, University of Helsinki, Helsinki, Finland; Department of Bacteriology and Immunology, University of Helsinki, Helsinki, Finland; Veterinary Biosciences, Faculty of Veterinary Medicine, University of Helsinki, Helsinki, Finland; Department of Virology, University of Helsinki and Helsinki University Hospital, Helsinki, Finland; Translational Immunology Research Program, University of Helsinki, Helsinki, Finland; Department of Neurology, Neurocenter, Helsinki University Hospital, Helsinki, Finland; Department of Bacteriology and Immunology, University of Helsinki, Helsinki, Finland; Clinical Microbiology, HUS Diagnostic Center, Helsinki University Hospital, Helsinki, Finland; Veterinary Biosciences, Faculty of Veterinary Medicine, University of Helsinki, Helsinki, Finland; Department of Otorhinolaryngology, Head and Neck Surgery, Helsinki University Hospital, Helsinki, Finland; Pediatric Cardiac and Transplantation Surgery Department, Helsinki Children's Hospital, University of Helsinki, Helsinki, Finland; Department of General Thoracic and Esophageal Surgery, Heart and Lung Center, Helsinki University Hospital and University of Helsinki, Helsinki, Finland; Department of General Thoracic and Esophageal Surgery, Heart and Lung Center, Helsinki University Hospital and University of Helsinki, Helsinki, Finland; Department of Pathology, University of Helsinki, Helsinki, Finland; Pathology, HUS Diagnostic Center, Helsinki University Hospital, Helsinki, Finland; Department of Pathology, University of Helsinki, Helsinki, Finland; Pathology, HUS Diagnostic Center, Helsinki University Hospital, Helsinki, Finland; Translational Immunology Research Program, University of Helsinki, Helsinki, Finland; Department of Neurology, Neurocenter, Helsinki University Hospital, Helsinki, Finland; Department of Virology, University of Helsinki and Helsinki University Hospital, Helsinki, Finland; Department of Virology, University of Helsinki and Helsinki University Hospital, Helsinki, Finland; Translational Immunology Research Program, University of Helsinki, Helsinki, Finland; Department of Bacteriology and Immunology, University of Helsinki, Helsinki, Finland; Clinical Microbiology, HUS Diagnostic Center, Helsinki University Hospital, Helsinki, Finland

**Keywords:** myasthenia gravis, thymus, thymoma, DNA viruses, parvovirus B19, herpesviruses

## Abstract

Myasthenia gravis (MG) is a rare autoimmune disorder characterized by muscle weakness resulting from autoantibody-mediated disruption of the neuromuscular junction. Notably, it is also frequently associated with thymic pathology. This study explores the relationship between MG and DNA viruses in the thymus, employing targeted next-generation sequencing and quantitative polymerase chain reaction to analyze thymic tissue samples from both patients with MG and healthy controls. We detected human herpes virus 6B and 7, Epstein-Barr virus, and parvovirus B19 (B19V) across various tissue groups. However, no significant enrichment of these viruses was observed in the thymic tissue of patients with MG.

## Teaser

DNA viruses show no direct association with thymic pathology in myasthenia gravis, making them unlikely primary triggers of the disease. However, their persistence in both myasthenia gravis and histologically normal thymus may have potential implications for thymic function.

Myasthenia gravis (MG) is a rare autoimmune disease characterized by the production of autoantibodies that target the nicotinic acetylcholine receptors (AChRs) or functionally related proteins (MuSK and LRP4), which disrupt neuromuscular transmission causing pronounced skeletal muscle weakness [1]. MG is a diverse condition in both clinical presentation and underlying pathophysiology. It comprises 3 main subgroups, each with distinct thymus involvement: (1) early-onset MG (EOMG), characterized by thymic follicular hyperplasia and symptom onset before age 50 years; (2) late-onset MG (LOMG), with less association to thymic pathologies and symptoms that emerge after age 50 years; and (3) thymoma-associated MG (TAMG), where MG is considered a paraneoplastic phenomenon. In both EOMG and TAMG, AChR autoantibody-producing ectopic germinal centers (eGCs) often develop within the thymus [1, 2]. While the significance of circulating anti-AChR autoantibodies in the pathogenesis of MG is well established, the underlying etiology of MG remains largely elusive.

Viral infections are generally recognized as potential triggers for autoimmunity. Chronic inflammation and Toll-like receptor activation in the thymus of patients with MG support the idea that a persistent viral infection could break the immune tolerance locally in genetically predisposed individuals [3]. While Epstein-Barr virus (EBV) and cytomegalovirus (CMV) have been investigated in MG, definitive evidence of their role in the pathogenesis is still lacking [3, 4]. Other human herpesviruses, such as HHV-6 and HHV-7, have been shown to replicate within the thymus [5]. More recently, parvovirus B19 (B19V), a single-stranded DNA virus with a narrow tropism for erythroid cells, has been linked to thymic hyperplasia in MG [6], and highlighted as plausible contributor to the formation of eGCs in TAMG [7].

No direct link between viral infections and MG has thus far been confirmed. Previous studies have focused only on 1 virus at a time and have used archival formalin-fixed tissue samples, which is a suboptimal material for viral detection. We decided to comprehensively assess the potential relationship between DNA viruses and thymic involvement in MG by employing targeted next-generation sequencing (NGS) for broad viral screening, followed by quantitative polymerase chain reaction (qPCR) analysis of B19V and 9 herpesviruses (herpes simplex virus 1 [HSV-1], HSV-2, varicella-zoster virus [VZV], EBV, CMV, HHV-6A, HHV-6B, HHV-7, and HHV-8) across a large collection of fresh thymic tissue. Additionally, we analyzed the B19V serostatus of patients using paired blood samples to identify those who had experienced a prior infection.

## METHODS

### Study Subjects and Samples

Thymic tissue samples were obtained from patients with MG and without MG undergoing thymectomy at Helsinki University Hospital (2020–2024). Control groups included thymic tissue from pediatric cardiac surgery patients (Ethical review board approval:HUS/124/2023 § 19/2023), adult thoracic surgery patients (Ethical review board approval: HUS/151/2022 § 37/2022), and tonsil tissue from patients without any known autoimmune disorders (Ethical review board approval: HUS/462/2021). Tissue samples were processed as either formalin-fixed paraffin-embedded (FFPE), directly frozen, or Optimal Cutting Temperature Compound (OCT)-embedded. Blood samples were collected at the time of surgery. Detailed protocols are provided in Supplementary Methods. All participants provided written informed consent.

### Detection of DNA Viruses by qPCR

The DNA extraction was performed with the QIAamp DNA Mini Kit (Qiagen), following the manufacturer's protocol for tissue extraction, except for the addition of a digestion step with collagenase and an increased volume of proteinase K [8]. To detect and quantify B19V DNA, a pan-B19V qPCR assay was employed, as previously described [9]. For detection of HSV-1, HSV-2, VZV, EBV, CMV, HHV-8, HHV-6A, HHV-6B, and HHV-7 a 3-tube multiplex qPCR assay was performed [10]. The human gene *RNase P* was quantified both as an internal control and to normalize the viral copy numbers per million cells across all the samples [9].

### B19V Serology

Serum samples were analyzed for acute and past infections of parvovirus B19V by IgM and IgG enzyme immunoassays using biotinylated virus-like particles of VP2 as antigens [11]. Epitope-type specific immunoglobulin G (IgG) towards linear peptide was also conducted to confirm prior immunity [12, 13].

### Laser Capture Microdissection

Three thymic samples from patients with EOMG with eGCs and 3 tonsil samples with the highest B19V DNA loads were selected for laser capture microdissection (LCM). Zeiss Membrane Slide 1.0 PEN were heat-treated and exposed to UV light for 30 minutes at a distance of 400 mm. From OCT blocks 12-μm thick serial sections were cut at −20°C, mounted on membrane slides, and stained with toluidine blue [14]. B-cell follicles, cortex, and medulla areas were dissected using a PALM Microbeam (Carl Zeiss MicroImaging GmbH) and collected in Zeiss Opaque Adhesive Cap tubes, then stored at −80°C.

### RNAscope In Situ Hybridization

RNAscope in situ hybridization (RISH) technology (ACD) was used to detect B19V DNA/RNA in 5-μm-thick FFPE thymus and tonsil tissue sections on glass slides (SuperFrost Plus). Detection was achieved using 20 pairs of double-Z oligonucleotide probes targeting the sense B19V *NS1* gene (probe-V-B19-NS1, 967–2215 bp; GenBank, NC 000883.2), as previously described [15]. Probes were amplified using RNAscope 2.5 HD Reagent Kit-RED (ACD) with a 7-minute Protease Plus incubation. Human *PPIB* and bacterial *dapB* probes (ACD) were used as positive and negative controls, respectively, with B19V PCR-negative tissues as additional controls.

### Statistical Analysis

Seroprevalences were compared using χ^2^ test. Pairwise comparisons of viral frequency employed Fisher exact test with Bonferroni correction. B19V viral loads were analyzed with Kruskal-Wallis test with Dunn post-hoc test. Significance was set at *P* < .05, and analyses were conducted in RStudio (version 2024.04.1 + 748).

## RESULTS

### Detection of DNA Viruses by NGS and Quantitative PCR

A pilot analysis was conducted on 16 DNA samples from fresh thymic tissue of patients with MG and controls to assess viral prevalence using targeted NGS with a custom viral DNA panel (Supplementary Methods and Supplementary Table 3). This analysis identified B19V and several herpesviruses, including HHV-6B and HHV-7, as the most prevalent viral agents, thereby suggesting them as potential candidates in the etiopathogenesis of MG. To validate these NGS findings, Pan-B19V qPCR and HERQ9 multiplex qPCR for herpesviruses were employed and subsequently applied to a larger sample cohort (Supplementary Table 4).

**Figure 1.**
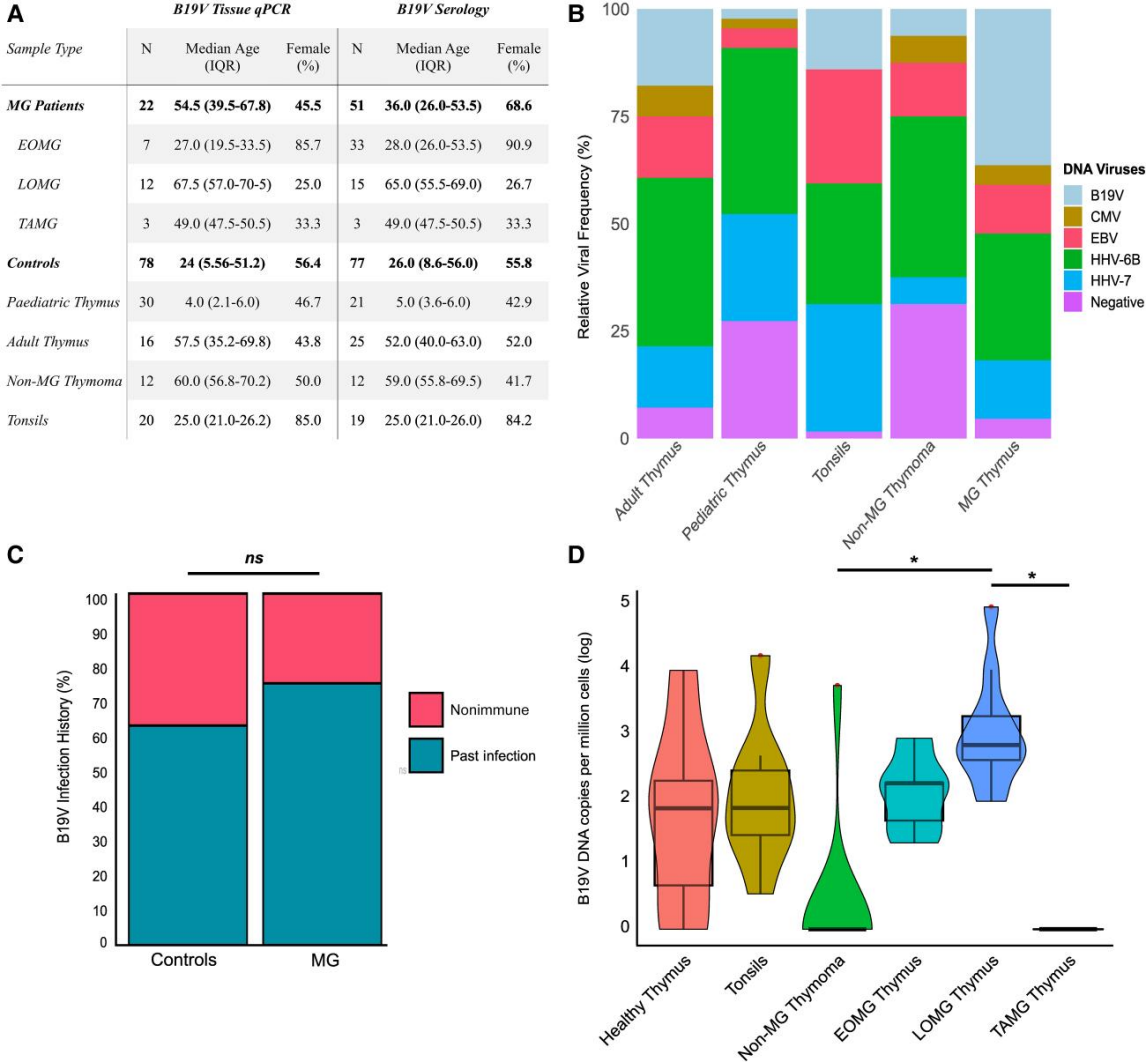
Comparative analysis of DNA virus presence in MG thymic tissues and controls, accompanied by serological screening for B19V in paired blood samples. *A*, Table of cohort characteristics. *B*, Relative frequency of DNA viruses across tissue groups. Viral prevalence was defined as the proportion of positive samples within each group, normalized by group size. The stacked bar plot allows for direct comparison across groups with varying sample sizes, with the “negative” group representing samples that tested PCR negative for all screened DNA viruses. B19V showed statistically higher prevalence in the MG group compared to pediatric thymus and thymoma, as well as in pediatric thymus compared to tonsils (*P* < .05). EBV was more frequently detected in tonsils than any other group (*P* < .05). HHV-7 was also significantly higher in tonsils compared any other tissue group (*P* < .05). No significant differences were observed for HSV-1, HSV-2, VZV, CMV, HHV-6A, HHV-6B, or HHV-8. *C*, Serological evaluation of B19V seroprevalence utilizing paired plasma samples from the original MG cohort and controls, supplemented by an archival cohort of plasma from patients with MG and controls. Pediatric samples were excluded from this analysis due to age bias. The median age of the MG group was 36 years (SD 18.3 years) (n = 51), whereas for the control group, it was 42.5 years (SD 19 years) (n = 56). No significant difference in the past B19V infection history between patients with MG and age-matched controls could be detected (*P* = .26). *D*, Box plot illustrating B19V copy numbers per 1 million cells (normalized for *RNase P*) in the different subsets of MG compared to control tissues. Significant differences in viral loads could be detected between the LOMG group and the thymoma and TAMG groups (*P* = .0002, *P* = .0105, respectively). The asterisks indicate statistical significance between groups. The box and whiskers represent the interquartile range (25th to 75th percentile), with the median marked by a line inside the box. The whiskers extend to 1.5 times the IQR, with data points outside this range considered outliers. Abbreviations: B19V, parvovirus B19; CMV, cytomegalovirus; EBV, Epstein-Barr virus; EOMG, early-onset MG; HHV, human herpesvirus; HSV, herpes simplex virus; LOMG, late-onset MG; MG, myasthenia gravis; ns, not significant; PCR, polymerase chain reaction; TAMG, thymoma-associated MG; VZV, varicella-zoster virus.

B19V DNA was most frequently detected in LOMG samples (11/12), with typical thymic involution. Lower frequencies were observed in EOMG (5/7), adult controls (6/16), and tonsillar tissues (9/20) (Supplementary Figure 3). Although the prevalence of B19V was higher in MG thymus tissues compared to controls, this difference reached statistical significance only when comparing MG thymus tissues to pediatric thymus and thymoma tissues. Importantly, there was no statistically significant difference observed between the MG thymus tissues and immunologically healthy adult control samples of a similar age (Figure 1*B*). The age comparison is crucial, as B19V IgG seroprevalence increases with age. Accordingly, B19V DNA was infrequently detected in pediatric thymic samples (1/30), consistent with a seroprevalence of 2 of 30. Thus, any differences in B19V prevalence between MG and pediatric thymic tissue reflects variations in infection history. Notably, B19V DNA was also infrequently detected in both TAMG and non-MG thymoma tissues (1/15), despite the advanced age and high seroprevalence in these individuals (13/15).

HHV-6B DNA showed a consistent genoprevalence across all examined thymic tissue groups (50%–76%) but was notably higher in tonsillar samples (90%). Similarly, HHV-7 DNA was present in thymic tissues at frequencies ranging from 8% to 57%, compared to 95% in tonsils. EBV DNA was infrequently detected in thymic tissues (5/22 MG, 2/12 non-MG thymoma, and 6/46 healthy thymus samples) and at low copy numbers, but was more commonly detected in tonsils (17/20) with higher copy numbers per million cells. CMV DNA was identified in 2 of 22 MG samples and 4 of 58 thymic controls (healthy and thymoma) but was absent in all tonsil samples (Figure 1*B*).

### B19V Serology and Viral Prevalence in Tissues of Seropositive Individuals

Serological testing of paired plasma samples revealed no significant difference in B19V seroprevalence between patients with MG and age- and sex-matched controls (*P* = .26; Figure 1*A* and *1C*). Archival plasma from a larger EOMG cohort also showed a subset of patients with MG with thymic pathologies, such as hyperplasia (8/23) and eGCs (5/23), who were seronegative for B19V (Supplementary Table 2). This indicates that B19V is not a prerequisite for the development of MG, or the thymic pathologies associated with the disease.

Among seropositive individuals for whom paired fresh tissues were available, qPCR confirmed B19V DNA in all cases except for TAMG and non-MG thymoma tissue. B19V viral copy numbers were similar between patients with EOMG and controls, but LOMG samples showed significantly higher viral loads compared to TAMG and non-MG thymoma tissues (Figure 1*D*).

### B19V DNA Persistence in Thymic Medulla But Absence in Autoantibody-Producing eGCs

**Figure 2.**
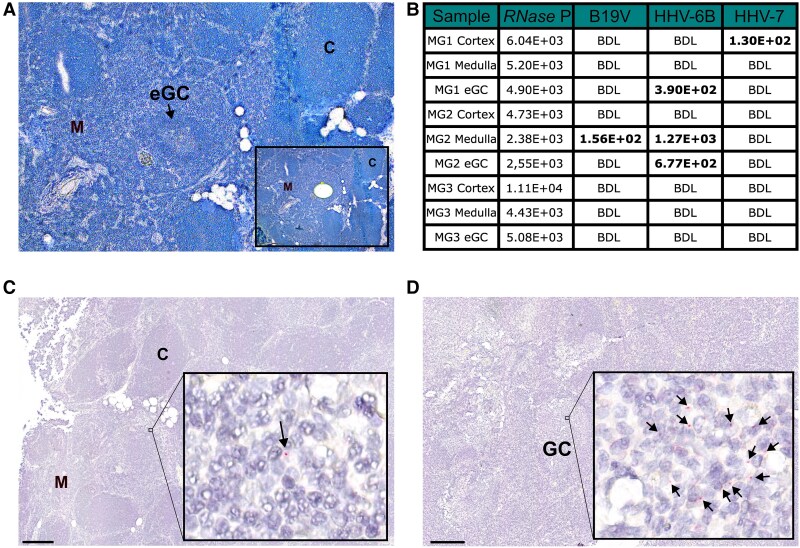
Laser capture microdissection and qPCR from regions of EOMG thymi, and RNAscope in situ hybridization of EOMG thymus and tonsil tissue from a control. *A*, Cryosections of EOMG thymi were stained with toluidine blue, followed by laser capture microdissection to isolate distinct regions: eGCs, medulla, and cortical areas, each collected into separate tubes (n = 3). *B*, Subsequent qPCR analysis targeting all herpesviruses and B19V was performed. *RNase P* values confirmed adequate tissue collection across all regions and samples. Notably, B19V-DNA detection was limited to the medullary region with the highest B19V copy numbers per 1 million cells (from whole tissue), whereas HHV-6B DNA was detected in both the medulla and eGCs of the tissue, and HHV-7 DNA in the cortex of a single sample. *C*, RISH for B19V nucleic acids on EOMG thymic tissue revealed sparse DNA-positive signals distributed throughout the tissue but localized within the medullary regions devoid of eGCs. The arrow indicates a positive B19V signal. *D*, B19V RISH on PCR-positive tonsillar control tissues demonstrated DNA-positive signals primarily within germinal centers. The arrows indicates positive B19V signals. Scale bar = 200 µm. Abbreviations: RISH, RNAscope in situ hybridization; B19V, parvovirus B19; C, cortex; eGC, ectopic germinal center; EOMG, early-onset myasthenia gravis; GC, germinal center; HHV, human herpesvirus; M, medulla; qPCR, quantitative polymerase chain reaction.

LCM was employed to isolate eGCs, medulla, and cortex from EOMG thymic tissues to explore the microanatomical persistence of B19V and herpesviruses (Figure 2*A*). Despite low B19V copy numbers in whole tissues, analysis of LCM-isolated areas revealed that B19V DNA was predominantly localized to the medulla, with no detectable presence in eGCs or cortex (Figure 2*B*), whereas HHV-6B was detected in both eGCs and medullary regions. Using RISH, we confirmed B19V-DNA was restricted to the medulla without colocalization with eGCs. Conversely, in tonsillar tissue, B19V DNA was detected primarily in GCs (Figure 2*C* and *2D*).

## DISCUSSION

The role of the thymus in the pathogenesis of MG has been extensively researched [2, 3, 4]. However, identifying the precise trigger that leads to thymic follicular hyperplasia-related MG has remained challenging. In this study, we employed a comprehensive approach utilizing fresh thymic tissues from both patients with MG and healthy controls to investigate a diverse array of DNA viruses and their role as potential contributors to MG. We observed the persistence of several DNA viruses within thymic tissues from individuals with normal thymic histology and those affected by MG. However, we did not find a relationship between any specific DNA virus and MG.

Previous research has suggested a possible productive infection of B19V in MG-associated thymic tissues [6, 7]. However, evidence supporting B19V replication in nonerythroid tissues remains limited. It has been shown that B19V can enter nonpermissive cells, but its potential for reactivation in previously healthy individuals has yet to be demonstrated [8]. To address these uncertainties, we conducted immunohistochemistry for B19V VP1/VP2 but detected no specific viral proteins in any thymic tissues (Supplementary Figure 4). Therefore, we conclude that the persistence of B19V within the thymus seems to be dormant, with no virion production or subsequent replication.

Our study has several strengths compared to previous reports [6, 7]. For viral DNA detection we used NGS and RT-PCR instead of nested PCR and used only fresh tissue samples with careful mitigation for environmental contamination. Moreover, we had access to paired blood samples, which allowed us to control for infection history. We also can conclude that residual circulating blood in the analyzed tissue samples is unlikely to account for the detected viruses, as plasma samples were predominantly negative for the virus (Supplementary Table 5). Seroprevalence of B19V increases with advancing age and, when we used an age-matched control group, we could not detect any difference in B19V-seropositivity rate between MG and controls. Our analysis of B19 serostatus indicated that the viral DNA persisted in thymic tissue of all previously infected individuals, except in thymoma cases, regardless of MG status. This finding suggests that thymoma tissue may have a distinctive environment that is less conducive to B19V persistence.

EBV was rarely detected in thymus, contradicting previous studies suggesting a possible role for EBV in MG pathogenesis [4]. The absence of EBV in thymic tissues, despite the presence of eGCs and B cells, is intriguing. Given the high seroprevalence of EBV, this raises the possibility that eGCs could originate from thymic B cells rather than peripheral B cells, suggesting a potential intrathymic protective mechanism against viral infection. Prevalence of the T-cell tropic HHV-6 in the thymus, on the other hand, raises concern for its potential reactivation and its impact on thymopoiesis, especially in immunocompromised hosts. HHV-6 has shown thymic depletion in animal models, suggesting that viral persistence could impair thymic output [5]. Our study identified HHV-6B and HHV-7 as viruses that frequently persist in the thymus of both patients with MG and healthy controls, emphasizing the need for further research into the relatively understudied impact of these viruses on the thymic microenvironment.

Although our findings do not link a particular DNA virus to MG, they highlight the susceptibility of the thymus to viral persistence, which may have broader implications for thymic function.

## Supplementary Material

jiae600_Supplementary_Data

## Supplementary Data

Supplementary materials are available at *The Journal of Infectious Diseases* online (http://jid.oxfordjournals.org/). Supplementary materials consist of data provided by the author that are published to benefit the reader. The posted materials are not copyedited. The contents of all supplementary data are the sole responsibility of the authors. Questions or messages regarding errors should be addressed to the author.

## Notes

***Author contributions.*** K. N., L. H., I. M. A., M. S. V., M. F. P., and E. K. contributed conceptualization and validation. K. N., L. H., I. M. A., P. D., M. I. M., L. H., and K. H. contributed methodology. K. N., L. H., and I. M. A. performed formal analysis and wrote the original draft. K. N., L. H., I. M. A., P. D., J. S., I. K. I., L. H., S. S., M. I. M., J. D., S. M. L., and M. F. P. performed investigations. M. I. M., I. K. I., K. N., S. S., S. M. L., M. F. P., L. H., J. R., A. H., P. T., L. M. A., J. D., M. S. V., and E. K. contributed resources. K. N., L. H., and J. S. performed data curation. K. N., L. H., I. M. A., J. S., M. I. M., I. K. I., M. N., S. S., S. M. L., M. F. P., K. H., J. R., A. H., P. T., M. S. V., and E. K. reviewed and edited the manuscript. K. N. performed visualization. M. I. M., S. M. L., K. H., M. S. V., M. F. P., and E. K. contributed supervision. K. N., M. F. P., and E. K. performed project administration. M. I. M., K. H., M. S. V., M. F. P., and E. K. contributed funding acquisition.

***Acknowledgments.*** We are grateful to the patients and healthy controls for their participation; without them, this study would not have been possible. We thank Marjo Rissanen for her help with sample processing. Additionally, we extend our thanks to Mikko Räsänen, Eva Sutinen, and Maarit Ylikulju for their contribution to patient procurement. The authors also thank the Genome Biology Unit, supported by HiLIFE and the Faculty of Medicine, University of Helsinki, and Biocenter Finland, for their imaging services.

***Financial support.*** This work was supported by the European Joint Programme on Rare Diseases and Research Council of Finland (grant number 308913 to E. K.); Doctoral Programme in Integrative Life Science, University of Helsinki (to K. N.); Finnish Medical Foundation (to M. F. P.); Life and Health Medical Support Association (to M. F. P. and M. S. V.); The Sigrid Jusélius Foundation (to M. S. V.); Helsinki University Hospital Research and Education Fund (to K. H.); Finska Läkaresällskapet (to K. H. and M. F. P.); Magnus Ehrnrooth Foundation (to K. H.); and Jane and Aatos Erkko Foundation (to K. H.).
