# Supplementary material for: Parvovirus B19 and Human Herpes Virus 6B and 7 Are Frequently Found DNA Viruses in the Human Thymus But Show No Definitive Link With Myasthenia Gravis
Source: J Infect Dis. 2024 Dec 5;231(4):e601–6. doi: 10.1093/infdis/jiae600 (PMC11998553; doi:10.1093/infdis/jiae600)
Supplement: jiae600_Supplementary_Data [file jiae600_supplementary_data.docx]

HHV-6B, HHV-7, and B19V Are Frequently Found DNA Viruses in the Human Thymus but Show No Definitive Link with Myasthenia Gravis

**Kirsten Nowlan, Leo Hannolainen, Irini M. Assimakopoulou, Pia Dürnsteiner, Joona Sarkkinen, Santeri Suokas, Lea Hedman, Pentti J. Tienari, Klaus Hedman, Mikael Niku, Leena-Maija Aaltonen, Antti Huuskonen, Jari V. Räsänen, Ilkka K. Ilonen, Mikko I. Mäyränpää, Johannes Dunkel, Sini M. Laakso, Maria Söderlund-Venermo, Maria F. Perdomo, Eliisa Kekäläinen**

**Supplementary Method**

**Mitigating risk for environmental contamination**

Surgical specimens from patients with MG and/or thymoma were obtained from the operating room and delivered to the pathology laboratory (HUS Diagnostic Center Pathology) in sterile, single-use plastic containers. Gross pathological evaluations were conducted in a fume hood on a clean, disposable barrier drape and dissections were performed using sterile, single-use scalpels. Portions of the MG and/or thymoma tissue not required for clinical diagnostics were designated for research analysis and placed in 50 mL factory-sterile Falcon tubes for transport to the clean room laminar for further processing. Adult thymus biopsies from thoracic surgery patients, tonsil tissues from patients without autoimmune disorders, and healthy paediatric thymus tissues from cardiac surgery patients were however collected directly from the operating room in sterile, single-use plastic containers and routed directly to the clean room laminar. All samples were further processed as per the experimental design (Supplementary Figure 1). This protocol maintained a strictly sterile environment, minimising the potential for cross-contamination. As a result, any viruses are likely to represent intrinsic tissue-associated viruses rather than contaminants from external sources.

###
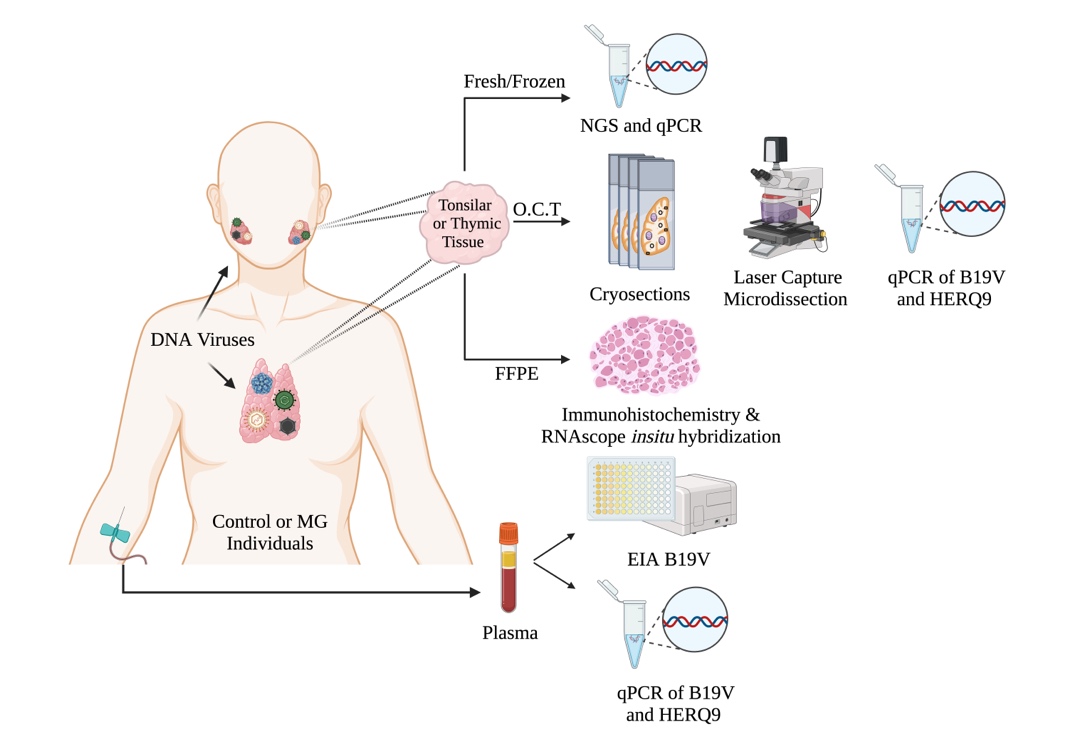


***Supplementary Figure 1****:* **Schematic representation of the study design**. Thymic and tonsillar tissues were processed into three types of preparations: formalin-fixed, paraffin-embedded (FFPE); OCT-embedded and rapidly frozen; and directly frozen at -80°C, before downstream analysis. Paired blood samples were obtained at the time of operation. This schematic was created with BioRender.com.

##### **Detection of DNA Viruses Using NGS**

DNA extracts were mechanically fragmented using a Covaris E220 instrument, with target size of 200 bp. The library preparation was performed using the KAPA Hyperplus kit (Roche) according to the manufacturer's protocol using unique double index adapters (Roche). For viral DNA capture, a custom panel of biotinylated RNA oligonucleotides (myBaits Custom DNA-Seq, Arbor Biosciences) was used [1]. Each individual sample underwent two rounds of hybridization, adhering to the manufacturer's recommendations for low-input DNA (MyBaits v5 kit Arbor Biosciences) and using KAPA Universal Enhancing Oligos ((Roche) to prevent non-specific binding. These RNA-probes had a length of 100 bp and were designed complementary to the complete sequences of 42 human DNA viruses with 2X tiling. Included in the design were parvovirus B19, cutavirus, torque teno viruses 1, 10, and 13, nine herpesviruses, polyomaviruses 1–13, hepatitis B virus, papillomavirus types 2, 6, 11, 16, 18, 21, 45, bocaviruses 1–4, simian virus 40, and variola viruses minor and major. The libraries were quantified using the KAPA Library Quantification Kit (Roche) and pooled for sequencing in NovaSeq 6000 (one lane, S4, PE151, Illumina). The libraries underwent 3 × 13–25 cycles of amplification, with clean-up using KAPA Pure Beads (Roche). Negative controls (PCR-grade water) were included in library preparation, enrichment, and sequencing.

##### **NGS data analysis**

The data analysis was done with TRACESPipeLite, a streamlined version of TRACESPipe [2]. The paired-end reads were trimmed and collapsed with AdapterRemoval, cutting ambiguous bases at the 5'/3' termini with quality scores below or equal to two. Reads shorter than 20 bases were discarded. FALCON-meta [3] was used to find the highest similar reference from the NCBI viral database. The reads were aligned with BWA [4] using a seed length of 1000 and a maximum diff of 0.01.  Read duplicates were removed with SAMtools [5] and the consensus sequences reconstructed with BCFtools [6]. The coverage profiles were created with BEDtools [7]. When in low breadth coverage (< 15 %), the individual reads were manually inspected and confirmed by BLAST. The pipeline is freely available, under MIT licence, at https://github.com/viromelab/TRACESPipeLite, along with the code (included in the TRACESPipeLite repository).

**Immunohistochemistry**

Immunohistochemical staining was performed according to the manufacturer's instructions on formalin-fixed paraffin-embedded (FFPE) sections of 3-µm-thick thymus and tonsil tissues using monoclonal antibodies against B19V VP1/VP2 (R92F6, Novocastra, Newcastle, UK). Protein staining was considered positive if granular brown reaction products could be observed in the nucleus. Placental tissue from B19V-infected individuals served as a positive control and PCR-negative tissue samples as negative controls [8]. All IHC stainings were performed at an accredited clinical pathology laboratory (HUS Diagnostic Center, Pathology).

### **Supplementary Results**

***Supplementary Table 1:*** **B19V qPCR results from thymoma tissue and adjacent adipose tissue.** *BDL= below detection limit*

| Sample | Sample Type | RNase P (copies/ul) | B19V per million cells | HHV-6B per million cells | HHV-7 per million cells |
| --- | --- | --- | --- | --- | --- |
| Thymus 45 | Thymoma tissue type A | 7.51e+05 | BDL | BDL | BDL |
| Thymus 45 | Adjacent adipose tissue | 2.52e+04 | 1.33e+04 | BDL | BDL |
| Thymus 36 | Thymoma tissue type B2 | 1.77e+06 | BDL | 4.68e+02 | BDL |
| Thymus 36 | Adjacent adipose tissue | 4.37e+04 | 2.19e+02 | BDL | BDL |
| Thymus 28 | Thymoma tissue type A | 1.62e+05 | BDL | 9.94e+01 | BDL |
| Thymus 28 | Adjacent adipose tissue | 9.35e+04 | 3.49e+01 | 4.02e+01 | 1.70e+01 |

Adipose tissue in the thymus as a reservoir of B19V DNA

The predominant composition of LOMG thymic tissue is mostly adipose, with small islets of thymic tissue, consistent with age-related thymic involution. Unlike EOMG, LOMG rarely exhibits thymic pathology [9]. Given that LOMG samples displayed the highest B19V copy number per million cells, this suggests that adipose tissue may serve as a potential reservoir for B19V persistence. Additionally, in a subset of thymoma samples, we dissected adjacent adipose tissue and processed it separately for qPCR (Supplementary Table 1). In seropositive individuals, B19V DNA was consistently detected in adipose tissue, while corresponding thymoma tissue from the same individuals tested negative by qPCR, regardless of thymoma subtype. These findings further support the hypothesis that adipose tissue may act as a reservoir for persistent B19V.


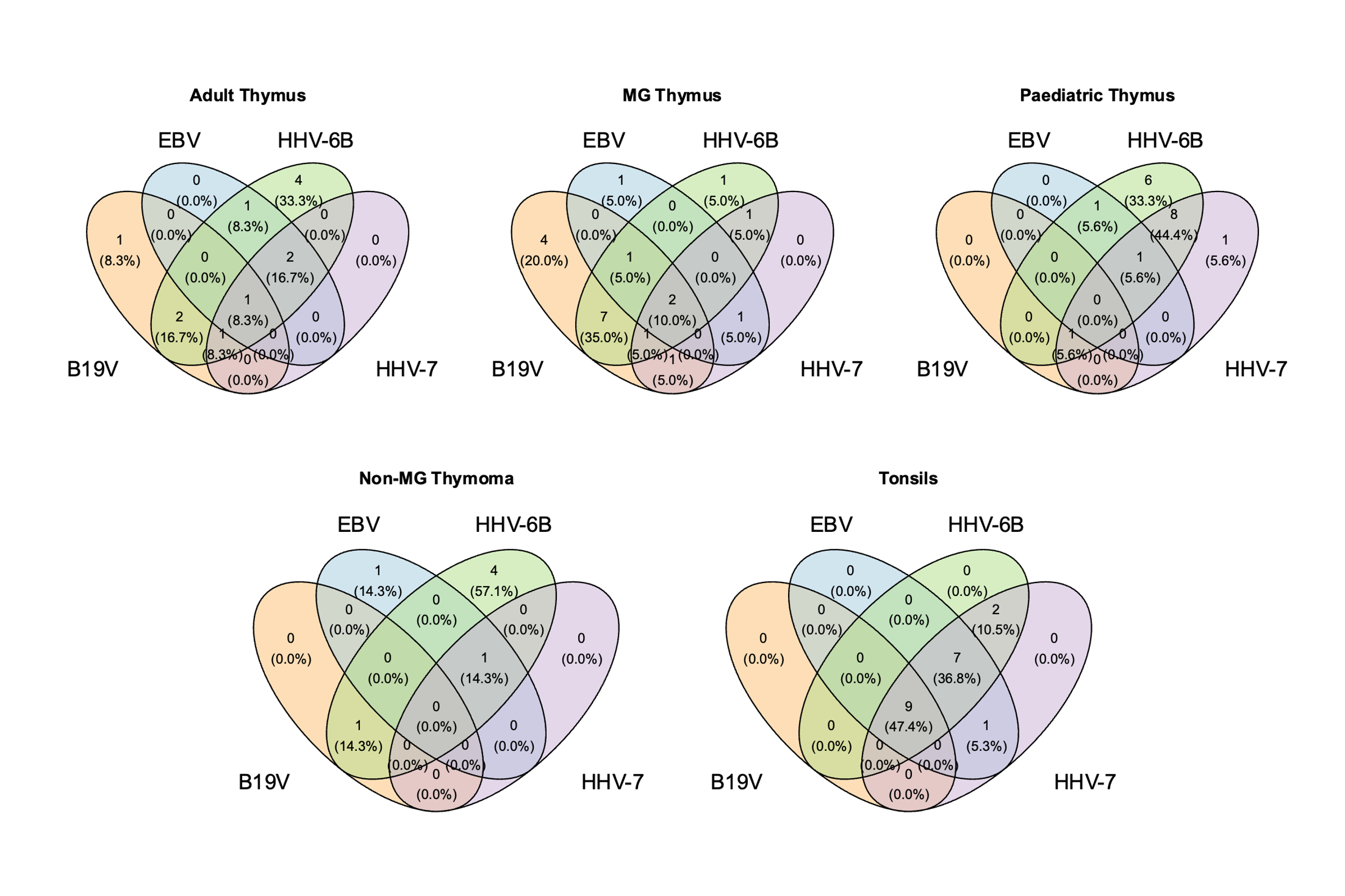


***Supplementary Figure 2****:* **Comparative analysis of DNA virus co-occurrence across tissue groups.** Each Venn diagram illustrates the overlap of viral presence within individual tissue samples for each group, highlighting instances where multiple viruses coexist in the same tissue. CMV was excluded from the visualisation due to its infrequent detection, enhancing clarity in visualizing the co-occurrence of the more prevalent viruses.

Viral Co-Occurrence Patterns Differ Across Tissue Groups

Tonsillar tissue demonstrated the highest prevalence of multiple viral co-detections, with nearly half of the samples showing concurrent presence of B19V, HHV-6B, HHV-7, and EBV, with the most frequent co-occurrence observed between HHV-6 and HHV-7. Similarly, in paediatric thymic tissue, HHV-6 was commonly detected in conjunction with HHV-7. However, this co-occurrence was less pronounced in MG, adult thymic tissue, and non-MG thymoma tissue, where HHV-6B was either detected alone or co-detected with B19V (Supplementary Figure 2).


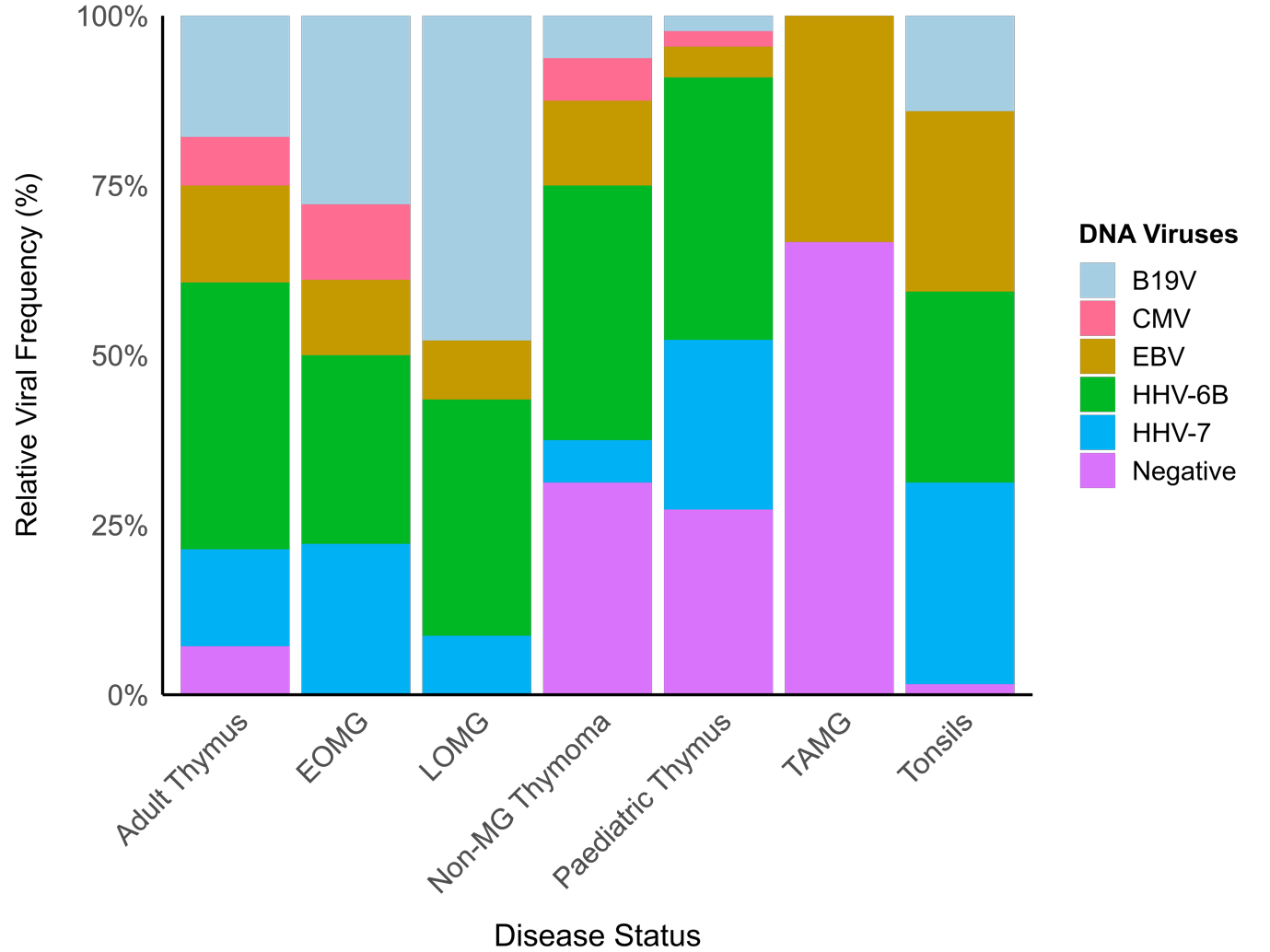


***Supplementary Figure 3****:* **Presence of DNA viruses in the subsets of MG patient thymic tissues and controls**. B) Stacked bar plot illustrating the relative frequency of viral detection in each tissue group. The 'Negative' group represents tissues PCR-Negative for all screened DNA viruses (herpesviruses and B19V).

LOMG Thymus Exhibits Highest Prevalence of B19V DNA

B19V DNA was most frequently detected in LOMG samples, which exhibited histological features of thymic involution and adipose deposition. In contrast, EOMG samples, characterised by thymic pathology including follicular hyperplasia and ectopic germinal centre formation, demonstrated slightly lower detection rates. Similarly, adult control thymic tissues, encompassing a broad age range with varying degrees of thymic involution, as well as tonsillar tissues, displayed lower detection levels (Supplementary Figure 3).


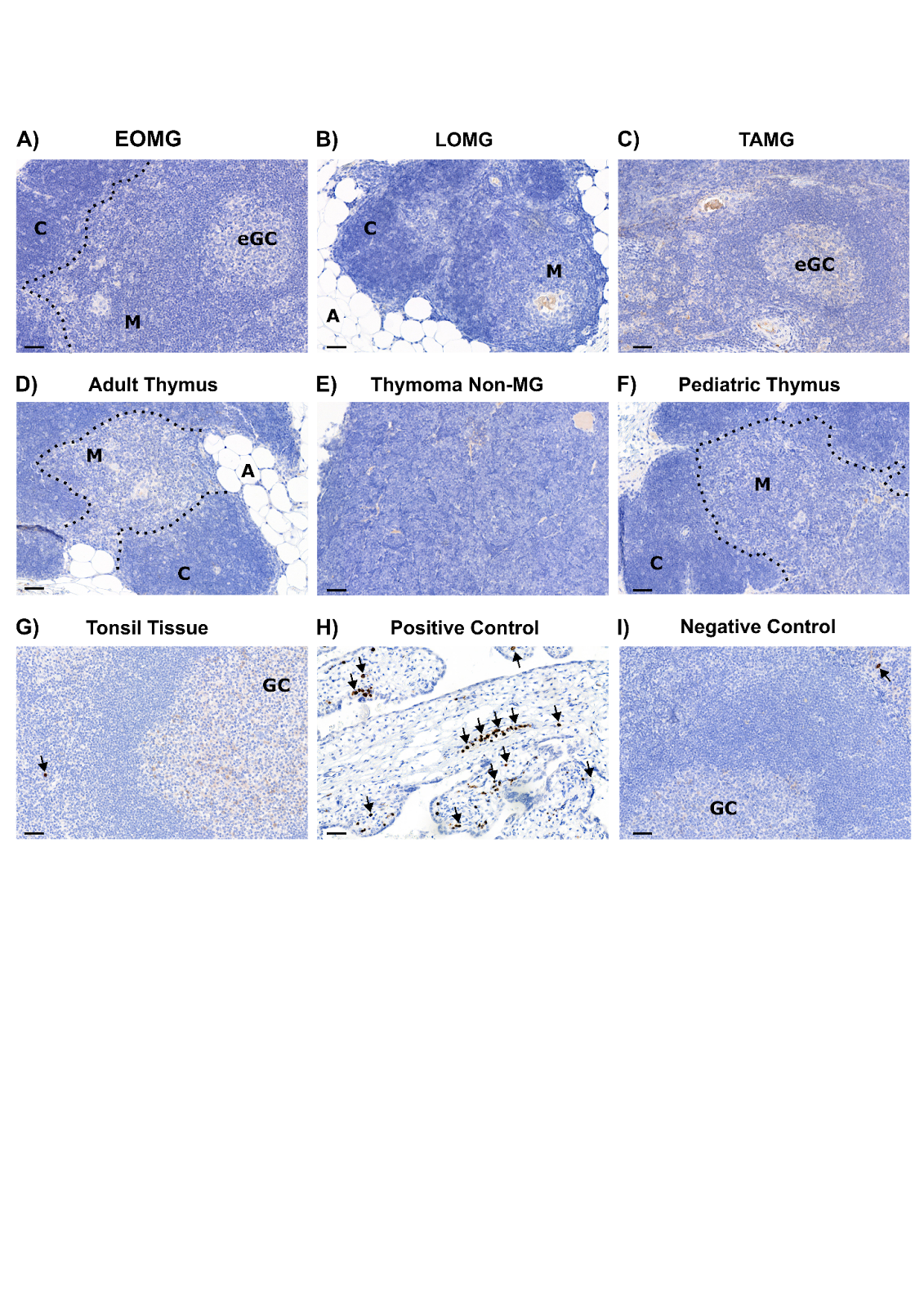


***Supplementary Figure 4:* Immunohistochemical Staining of B19V VP1/VP2 in Various Subsets of MG Thymic Tissues and Control Tissues.** A-F) B19V PCR-positive thymus tissue from seropositive individuals. A) Hyperplastic thymic tissue from an EOMG patient with eGCs present within the tissue. B) Involuted thymic tissue from a LOMG patient, in which thymic islets within adipose tissue are evident. C) Thymoma tissue from a patient with TAMG and eGCs present within the tissue. D) Healthy thymic tissue from an adult control sample which was macroscopically involuted, i.e thymic islets surrounded by adipose tissue. E) Thymoma tissue from a patient that does not have MG. F) Healthy thymic tissue from a paediatric individual, with little to no involution evident. B19V VP1/VP2 antigen staining was negative in all thymic tissues. G) B19V PCR-positive palatine tonsil tissue from an immunologically healthy B19V seropositive individual and genopositive. A dark nuclear signal could be detected in a few cells within the whole tissue section. H) Positive control placental tissue from an individual with an active B19V infection. Dark nuclear signals can be seen in multiple cells within the tissue. I)  B19V PCR-negative control palatine tonsil tissue from a seronegative individual; sparse dark nuclear signals can also be seen in a pattern similar to that of the qPCR-positive tonsil, indicating that this signal is likely to be nonspecific. Tissue images are 20X and the scale bar is 50µm. M = medulla, C = cortex, A= adipose, eGC = ectopic germinal centre, GC= germinal centre.

Detecting B19V VP1/VP2 Proteins with Immunohistochemistry

To understand whether the persistent B19V infection might manifest as a productive tissue infection, we employed immunohistochemistry (IHC) to identify active production of B19V VP1/VP2 proteins. We detected faint nonspecific signals that displayed a non-nuclear staining pattern and were found across all tissues, irrespective of seropositivity or seronegativity. No distinctly positive cells expressing B19V VP1/VP2 were evident in any thymic tissues when compared to a positive control sample. Likewise, the tonsil tissue exhibited one or two darker stains that could also be observed in the PCR-negative control tonsil tissue, indicating they are nonspecific signals by Xu et al. [8] (Supplementary Figure 4). Non-specific staining using this antibody has also been observed Our results are consistent with persisting B19V DNA in the thymus but without production of viral structural proteins.

**Supplementary Data**

***Supplementary Table 2****:* Patient characteristics from extended cohort of individuals with MG

| **Sample** | **Age Group (y)** | **Sex** | **MG**  **Type** | **B19V Serostatus** | **eGCs** | **Hyperplasia** |
| --- | --- | --- | --- | --- | --- | --- |
| MGO1 | 15-30 | F | EOMG | Non-Immune | Yes | Yes |
| MG02 | 15-30 | F | EOMG | Non-Immune | Yes | Yes |
| MG03 | 15-30 | F | EOMG | Non-Immune | Unknown | Unknown |
| MG04 | 15-30 | F | EOMG | Non-Immune | Yes | Yes |
| MG05 | 15-30 | F | EOMG | Non-Immune | Yes | Yes |
| MG06 | 30-50 | F | EOMG | Non-Immune | Unknown | Unknown |
| MG07 | 30-50 | F | EOMG | Non-Immune | Yes | Yes |
| MG08 | 30-50 | F | EOMG | Non-Immune | No | Yes |
| MG09 | >50 | M | LOMG | Non-Immune | Unknown | Unknown |
| MG10 | >50 | F | LOMG | Non-Immune | Yes | No |
| MG11 | 15-30 | F | EOMG | Past B19V infection | Unknown | Unknown |
| MG12 | 15-30 | F | EOMG | Past B19V infection | Unknown | Unknown |
| MG13 | 15-30 | F | EOMG | Past B19V infection | Yes | Yes |
| MG14 | 15-30 | F | EOMG | Past B19V infection | Yes | Yes |
| MG15 | 15-30 | F | EOMG | Past B19V infection | Unknown | Unknown |
| MG16 | 15-30 | F | EOMG | Past B19V infection | Yes | Yes |
| MG17 | 15-30 | F | EOMG | Past B19V infection | Yes | Yes |
| MG18 | 15-30 | F | EOMG | Past B19V infection | Yes | Yes |
| MG19 | 15-30 | F | EOMG | Past B19V infection | No | Yes |
| MG20 | 30-50 | F | EOMG | Past B19V infection | Yes | Yes |
| MG21 | 30-50 | F | EOMG | Past B19V infection | Unknown | Unknown |
| MG22 | 30-50 | F | EOMG | Past B19V infection | Yes | Yes |
| MG23 | 30-50 | F | EOMG | Past B19V infection | Yes | Yes |
| MG24 | 30-50 | F | EOMG | Past B19V infection | No | Yes |
| MG25 | 30-50 | F | EOMG | Past B19V infection | Unknown | Unknown |
| MG26 | 30-50 | F | EOMG | Past B19V infection | Yes | Yes |
| MG27 | 30-50 | M | EOMG | Past B19V infection | Unknown | Unknown |
| MG28 | 30-50 | M | EOMG | Past B19V infection | Unknown | Unknown |
| MG29 | >50 | M | LOMG | Past B19V infection | Unknown | Unknown |

***Supplementary Table 3****:* Breadth coverage of DNA viruses for pilot study of 16 samples by targeted NGS screening.

| **Sample** | **Age**  **Group (y)** | **Sex** | **MG** | **MG**  **Type** | **B19V** | **HHV-6B** | **HHV-7** | **TTV** | **MCPyV** |
| --- | --- | --- | --- | --- | --- | --- | --- | --- | --- |
| Thymus 1 | 30-50 | M | 1 | EOMG | - | - | - | - | - |
| Thymus 2 | 30-50 | M | 1 | EOMG | 93 | - | - | - | - |
| Thymus 3 | >50 | F | 0 | NA | - | - | - | - | - |
| Thymus 4 | >50 | M | 0 | NA | - | 7 | - | - | - |
| Thymus 5 | 15-30 | F | 1 | EOMG | 11 | - | - | - | - |
| Thymus 6 | >50 | F | 0 | NA | - | - | - | - | - |
| Thymus 7 | >50 | F | 1 | LOMG | 14 | - | - | - | - |
| Thymus 8 | >50 | M | 1 | LOMG | 18 | 6 | - | - | - |
| Thymus 9 | 15-30 | F | 1 | EOMG | 23 | - | - | - | - |
| Thymus 10 | >50 | M | 1 | LOMG | 99 | 14 | 13 | - | - |
| Thymus 11 | >50 | F | 0 | NA | - | - | - | - | - |
| Thymus 12 | >50 | F | 0 | NA | - | - | - | - | - |
| Thymus 13 | >50 | M | 0 | NA | 99 | 46 | 6,7 | 6,6 | - |
| Thymus 14 | 30-50 | F | 0 | NA | 94 | 7 | - | - | 3 |
| Thymus 15 | >50 | M | 1 | TAMG | 32 | - | - | - | - |
| Thymus 16 | 30-50 | F | 0 | NA | 92 | 14 | 1 | - | - |

***Supplementary Table 4****:* Cohort characteristics, and qPCR detection of DNA viruses from fresh tissue samples

| **Sample** | **Age Group (y)** | **Sex** | **Sample Group** | **Subset** | **B19V**  **Serostatus** | **B19V**  **DNA** | **EBV**  **DNA** | **CMV**  **DNA** | **HHV-6B DNA** | **HHV-7 DNA** | **Histology** |
| --- | --- | --- | --- | --- | --- | --- | --- | --- | --- | --- | --- |
| Thymus 4 | >50 | M | Control | Adult healthy | Non-Immune |  |  |  | X |  | Involution |
| Thymus 3 | >50 | F | Control | Adult healthy | Non-Immune |  |  |  | X |  | Involution |
| Thymus 16 | 30-50 | F | Control | Adult healthy | Past B19 infection | X |  |  | X |  | Involution |
| Thymus 13 | >50 | M | Control | Adult healthy | Past B19 infection | X | Not-tested | Not-tested | Not-tested | Not-tested | Involution |
| Thymus 52 | 30-50 | F | Control | Adult healthy | Non-Immune |  | X |  | X | X | Involution |
| Thymus 23 | >50 | F | Control | Adult healthy | Non-Immune |  |  |  | X |  | Involution |
| Thymus 38 | >50 | F | Control | Adult healthy | Past B19 infection | X |  | X | X | X | Involution |
| Thymus 47 | 15-30 | M | Control | Adult healthy | Non-Immune |  | X |  | X | X | Involution |
| Thymus 54 | 30-50 | F | Control | Adult healthy | Past B19 infection | X |  |  |  |  | Involution |
| Thymus 31 | 15-30 | F | Control | Adult healthy | Non-Immune |  |  |  |  |  | Involution |
| Thymus 63 | 30-50 | M | Control | Adult healthy | Unknown |  |  |  |  |  | Involution |
| Thymus 65 | >50 | M | Control | Adult healthy | Past B19 infection | X | X |  | X | X | Involution |
| Thymus 41 | >50 | M | Control | Adult healthy | Non-Immune |  |  |  | X |  | Involution |
| Thymus 81 | >50 | M | Control | Adult healthy | Unknown |  | X |  | X |  | Involution |
| Thymus83 | 15-30 | M | Control | Adult healthy | Unknown |  |  |  |  |  | Involution |
| Thymus82 | >50 | M | Control | Adult healthy | Unknown | X |  |  | X |  | Involution |
| Thymus 5 | 15-30 | F | MG | EOMG | Past B19 infection | X |  | X | X |  | Hyperplasia with eGCS |
| Thymus 1 | 30-50 | M | MG | EOMG | Non-Immune |  |  | X | X | X | Hyperplasia |
| Thymus 9 | 15-30 | F | MG | EOMG | Past B19 infection | X |  |  | X |  | Hyperplasia with eGCS |
| Thymus 17 | 15-30 | F | MG | EOMG | Past B19 infection | X |  |  |  | X | Hyperplasia with eGCS |
| Thymus 33 | >50 | F | MG | EOMG | Past B19 infection | X | X |  | X | X | eGCS |
| Thymus 53 | 15-30 | F | MG | EOMG | Non-Immune |  | X |  |  | X | Hyperplasia |
| Thymus 25 | 15-30 | F | MG | EOMG | Past B19 infection | X |  |  | X |  | Hyperplasia with eGCS |
| Thymus 8 | >50 | M | MG | LOMG | Past B19 infection | X |  |  | X |  | Involution |
| Thymus 7 | >50 | F | MG | LOMG | Past B19 infection | X |  |  |  |  | Involution |
| Thymus 10 | >50 | M | MG | LOMG | Past B19 infection | X |  |  | X |  | Involution |
| Thymus 2 | 30-50 | M | MG | LOMG | Past B19 infection | X |  |  | X |  | Involution |
| Thymus 42 | >50 | M | MG | LOMG | Past B19 infection | X | X |  | X |  | Involution |
| Thymus 30 | >50 | M | MG | LOMG | Past B19 infection | X |  |  | X |  | Involution |
| Thymus 62 | >50 | M | MG | LOMG | Past B19 infection | X |  |  |  |  | Involution |
| Thymus 27 | >50 | F | MG | LOMG | Past B19 infection | X | X |  | X | X | Involution |
| Thymus 70 | >50 | F | MG | LOMG | Non-Immune |  |  |  | X |  | Involution |
| Thymus 54 | >50 | M | MG | LOMG | Past B19 infection | X |  |  |  |  | Involution |
| Thymus 48 | >50 | M | MG | LOMG | Past B19 infection | X |  |  |  |  | Involution |
| Thymus 37 | 30-50 | M | MG | LOMG | Past B19 infection | X |  |  | X | X | Involution |
| Thymus 18 | <15 | F | Control | Ped healthy | Unknown |  |  |  |  |  | Normal |
| Thymus 61 | <15 | M | Control | Ped healthy | Unknown |  |  |  |  |  | Normal |
| Thymus 76 | <15 | M | Control | Ped healthy | Unknown |  |  |  |  |  | Normal |
| Thymus 69 | <15 | M | Control | Ped healthy | Unknown |  |  |  |  |  | Normal |
| Thymus 39 | <15 | F | Control | Ped healthy | Unknown |  |  |  | X |  | Normal |
| Thymus 79 | <15 | M | Control | Ped healthy | Unknown |  |  |  |  |  | Normal |
| Thymus 72 | <15 | F | Control | Ped healthy | Unknown |  |  |  |  |  | Normal |
| Thymus 21 | <15 | F | Control | Ped healthy | Unknown |  |  |  |  |  | Normal |
| Thymus 49 | <15 | F | Control | Ped healthy | Unknown |  |  |  |  |  | Normal |
| Thymus 74 | <15 | F | Control | Ped healthy | Non-Immune |  | X |  | X | X | Normal |
| Thymus 29 | <15 | F | Control | Ped healthy | Non-Immune |  |  |  |  |  | Normal |
| Thymus 68 | <15 | M | Control | Ped healthy | Non-Immune |  |  |  | X | X | Normal |
| Thymus 64 | <15 | F | Control | Ped healthy | Non-Immune |  |  |  | X | X | Normal |
| Thymus 43 | <15 | F | Control | Ped healthy | Non-Immune |  |  |  | X |  | Normal |
| Thymus 55 | <15 | M | Control | Ped healthy | Past B19 infection | X |  |  | X | X | Normal |
| Thymus 66 | <15 | F | Control | Ped healthy | Non-Immune |  |  |  | X |  | Normal |
| Thymus 19 | <15 | M | Control | Ped healthy | Non-Immune |  |  |  | X | X | Normal |
| Thymus 34 | <15 | M | Control | Ped healthy | Non-Immune |  |  |  |  |  | Normal |
| Thymus 50 | <15 | M | Control | Ped healthy | Non-Immune |  |  |  | X |  | Normal |
| Thymus 78 | <15 | M | Control | Ped healthy | Non-Immune |  |  |  |  |  | Normal |
| Thymus 80 | <15 | M | Control | Ped healthy | Non-Immune |  |  |  | X |  | Normal |
| Thymus 75 | <15 | F | Control | Ped healthy | Non-Immune |  |  |  | X |  | Normal |
| Thymus 24 | <15 | F | Control | Ped healthy | Non-Immune |  |  |  | X | X | Normal |
| Thymus 77 | <15 | F | Control | Ped healthy | Non-Immune |  |  |  | X | X | Normal |
| Thymus 59 | <15 | M | Control | Ped healthy | Non-Immune |  |  |  | X | X | Normal |
| Thymus 73 | <15 | M | Control | Ped healthy | Non-Immune |  |  |  |  |  | Normal |
| Thymus 32 | <15 | F | Control | Ped healthy | Non-Immune |  |  |  | X | X | Normal |
| Thymus 46 | <15 | M | Control | Ped healthy | Past B19 infection |  |  |  | X | X | Normal |
| Thymus 71 | <15 | M | Control | Ped healthy | Unknown |  | X |  | X |  | Normal |
| Thymus 67 | <15 | M | Control | Ped healthy | Unknown |  |  | X |  | X | Normal |
| Thymus 35 | <15 | M | Control | Ped healthy | Non-Immune | Not-tested | Not-tested | Not-tested | Not-tested | Not-tested | Normal |
| Thymus 44 | <15 | M | Control | Ped healthy | Non-Immune | Not-tested | Not-tested | Not-tested | Not-tested | Not-tested | Normal |
| Thymus 15 | >50 | M | MG | TAMG | Past B19 infection |  |  |  |  |  | Thymoma  B1 |
| Thymus 51 | 30-50 | M | MG | TAMG | Past B19 infection |  | X |  |  |  | Thymoma  B2/B3 |
| Thymus 60 | 30-50 | F | MG | TAMG | Past B19 infection |  |  |  |  |  | Thymoma  B1/B2 |
| Thymus 57 | >50 | M | Control | Thymoma | Past B19 infection |  |  |  |  |  | Thymoma  B3 |
| Thymus 12 | >50 | F | Control | Thymoma | Unknown |  | X |  | X | X | Thymoma  Micronodular |
| Thymus 6 | >50 | F | Control | Thymoma | Past B19 infection |  |  |  | X |  | Thymoma  AB |
| Thymus 11 | >50 | F | Control | Thymoma | Past B19 infection |  |  |  | X |  | Thymoma  B1 |
| Thymus 14 | 30-50 | F | Control | Thymoma | Past B19 infection | X |  | X | X |  | Thymoma  AB |
| Thymus 58 | >50 | F | Control | Thymoma | Past B19 infection |  |  |  |  |  | Thymoma  AB |
| Thymus 26 | >50 | F | Control | Thymoma | Past B19 infection |  |  |  |  |  | Thymoma  AB |
| Thymus 20 | >50 | M | Control | Thymoma | Non-Immune |  |  |  |  |  | Thymoma  B1 |
| Thymus 45 | >50 | M | Control | Thymoma | Past B19 infection |  |  |  |  |  | Thymoma  A |
| Thymus 40 | >50 | M | Control | Thymoma | Past B19 infection |  | X |  |  |  | Thymoma  AB |
| Thymus 36 | >50 | M | Control | Thymoma | Past B19 infection |  |  |  | X |  | Thymoma  B2 |
| Thymus 22 | 30-50 | M | Control | Thymoma | Past B19 infection | Not-tested | Not-tested | Not-tested | Not-tested | Not-tested | Thymoma  B2/B3 |
| Thymus 28 | >50 | M | Control | Thymoma | Past B19 infection |  |  |  | X |  | Thymoma  A |
| Tonsil 1 | 15-30 | F | Control | Tonsil | Past B19 infection | X | X |  | X | X | Normal |
| Tonsil 2 | 15-30 | F | Control | Tonsil | Non-Immune |  | X |  | X | X | Normal |
| Tonsil 3 | 15-30 | F | Control | Tonsil | Past B19 infection | X | X |  | X | X | Normal |
| Tonsil 4 | 15-30 | F | Control | Tonsil | Past B19 infection | X | X |  | \| X \| \| --- \| | X | Normal |
| Tonsil 5 | 15-30 | F | Control | Tonsil | Past B19 infection | X | X |  | X | X | Normal |
| Tonsil 6 | 15-30 | F | Control | Tonsil | Non-Immune |  | X |  | X | X | Normal |
| Tonsil 7 | 30-50 | F | Control | Tonsil | Non-Immune |  | X |  |  | X | Normal |
| Tonsil 8 | 15-30 | F | Control | Tonsil | Non-Immune |  |  |  | X | X | Normal |
| Tonsil 9 | 15-30 | F | Control | Tonsil | Non-Immune |  | X |  | X | X | Normal |
| Tonsil 10 | 15-30 | F | Control | Tonsil | Non-Immune |  | X |  | X | X | Normal |
| Tonsil 11 | 30-50 | F | Control | Tonsil | Past B19 infection | X | X |  | X | X | Normal |
| Tonsil 12 | 15-30 | M | Control | Tonsil | Non-Immune |  |  |  |  |  | Normal |
| Tonsil 13 | 30-50 | M | Control | Tonsil | Past B19 infection | X | X |  | X | X | Normal |
| Tonsil 14 | 15-30 | F | Control | Tonsil | Non-Immune |  | X |  | X | X | Normal |
| Tonsil 15 | 15-30 | M | Control | Tonsil | Past B19 infection | X | X |  | X | X | Normal |
| Tonsil 16 | 30-50 | F | Control | Tonsil | Unknown | X | X |  | X | X | Normal |
| Tonsil 17 | 15-30 | F | Control | Tonsil | Non-Immune |  |  |  | X | X | Normal |
| Tonsil 18 | 15-30 | F | Control | Tonsil | Past B19 infection | X | X |  | X | X | Normal |
| Tonsil 19 | 15-30 | F | Control | Tonsil | Non-Immune |  | X |  | X | X | Normal |
| Tonsil 20 | 15-30 | F | Control | Tonsil | Non-Immune |  | X |  | X | X | Normal |

***Supplementary Table 5****:* qPCR detection of DNA viruses from plasma samples from MG

| \| **Sample ID** \| \| --- \| | **RNaseP copies/µL** | **B19V copies/µL** | **EBV copies/µL** | **CMV copies/µL** | **HHV-6B copies/µL** | **HHV-7 copies/µL** |
| --- | --- | --- | --- | --- | --- | --- | --- |
| Thymus 4 | 1,01E+03 | - | - | - | - | - |
| Thymus 3 | 8,74E+02 | - | - | - | - | - |
| Thymus 16 | 2,88E+03 | - | - | - | - | - |
| Thymus 13 | 7,28E+01 | - | - | - | - | - |
| Thymus 23 | 4,49E+01 | - | - | - | - | - |
| Thymus 38 | 1,31E+03 | - | - | - | - | - |
| Thymus 47 | 1,83E+02 | - | - | - | - | - |
| Thymus 54 | 6,97E+02 | - | - | - | - | - |
| Thymus 31 | 1,24E+03 | - | - | - | - | - |
| Thymus 63 | 1,43E+03 | - | - | - | - | - |
| Thymus 65 | 6,56E+02 | - | - | - | - | - |
| Thymus 41 | 2,82E+03 | - | - | - | - | - |
| Thymus 81 | 3,80E+02 | - | - | - | - | - |
| Thymus83 | 6,89E+03 | - | - | - | - | - |
| Thymus82 | 5,67E+02 | - | - | - | - | - |
| Thymus 5 | 2,07E+03 | - | - | - | - | - |
| Thymus 1 | 1,30E+03 | - | - | - | - | - |
| Thymus 9 | 2,24E+03 | - | - | - | - | - |
| Thymus 17 | 7,81E+01 |  |  |  | 2,34E+00 |  |
| Thymus 33 | 2,28E+02 | - | - | - | - | - |
| Thymus 53 | 5,47E+02 | - | - | - | - | - |
| Thymus 25 | 2,11E+02 | - | - | - | - | - |
| Thymus 8 | 2,56E+03 | - | - | - | - | - |
| Thymus 7 | 1,21E+02 | - | - | - | 2,03E+00 | - |
| Thymus 10 | 1,04E+03 | - | - | - | - | 2,26E+00 |
| Thymus 2 | 1,49E+02 | - | - | - | - | - |
| Thymus 42 | 1,31E+02 | - | - | - | - | - |
| Thymus 30 | 2,25E+02 | - | - | - | - | - |
| Thymus 62 | 6,25E+01 |  |  |  |  |  |
| Thymus 27 | 4,82E+02 | - | - | - | - | - |
| Thymus 70 | 1,27E+02 | - | 2,03E+00 | - | - | - |
| Thymus 54 | 2,66E+02 | - | - | - | - | - |
| Thymus 48 | 4,85E+01 | - | - | - | - | - |
| Thymus 37 | 2,75E+02 | - | - | - | - | - |
| Thymus 15 | 5,68E+02 | - | - | - | - | - |
| Thymus 51 | 8,52E+01 | - | - | - | - | - |
| Thymus 60 | 9,78E+02 | - | - | - | - | - |
| Thymus 57 | 4,68E+02 | - | - | - | - | - |
| Thymus 6 | 1,31E+03 | - | - | - | - | - |
| Thymus 11 | 3,10E+03 | - | - | - | - | - |
| Thymus 14 | 1,29E+03 | - | - | - | - | - |
| Thymus 58 | 1,74E+03 | - | - | - | - | - |
| Thymus 26 | 1,85E+01 | - | - | - | - | - |
| Thymus 52 | 9,83E+01 | - | - | - | - | - |
| Thymus 20 | 1,12E+03 | - | - | - | - | - |
| Thymus 45 | 1,25E+03 | - | - | - | - | - |
| Thymus 40 | 1,29E+01 | - | - | - | - | - |
| Thymus 36 | 8,00E+01 | - | - | - | - | - |
| Thymus 22 | 9,50E+01 | - | - | - | - | - |
| Thymus 28 | 9,35E+00 | - | - | - | - | - |

### **Supplementary References**

1. Toppinen M, Pratas D, Väisänen E et al. The landscape of persistent human DNA viruses in femoral bone. Forensic Sci Int Genet 2020; 48: 102353.
2. Pratas D, Toppinen M, Pyöriä L et al. A hybrid pipeline for reconstruction and analysis of viral genomes at multi-organ level. Gigascience 2020; 9.
3. Pratas D, Hosseini M, Grilo G et al. Metagenomic Composition Analysis of an Ancient Sequenced Polar Bear Jawbone from Svalbard. Genes 2018; 9.
4. Li H, Durbin R. Fast and accurate long-read alignment with Burrows-Wheeler transform. Bioinformatics 2010; 26: 589–595.
5. Li H, Handsaker B, Wysoker A et al. The Sequence Alignment/Map format and SAMtools. Bioinformatics 2009; 25: 2078–2079.
6. Li H. A statistical framework for SNP calling, mutation discovery, association mapping and population genetical parameter estimation from sequencing data. Bioinformatics 2011; 27: 2987–2993.
7. Quinlan AR. BEDTools: The Swiss-Army Tool for Genome Feature Analysis. Curr Protoc Bioinformatics 2014; 47: 11.12.1–34.
8. Xu M, Leskinen K, Gritti T *et al.* Prevalence, Cell Tropism, and Clinical Impact of Human Parvovirus Persistence in Adenomatous, Cancerous, Inflamed, and Healthy Intestinal Mucosa. Front Microbiol 2022; 13: 914181.
9. Sarkkinen J, Dunkel J, Tuulasvaara A *et al.* Ectopic germinal centers in the thymus accurately predict prognosis of myasthenia gravis after thymectomy. Mod Pathol 2022; 1–7.
